# Chronic limb threatening ischemia and diabetes mellitus: the severity of tibial atherosclerosis and outcome after infrapopliteal revascularization

**DOI:** 10.1177/1457496920968679

**Published:** 2020-11-23

**Authors:** Veerakaisa Koivunen, Markus Juonala, Kimmo Mikkola, Harri Hakovirta

**Affiliations:** Faculty of Medicine Turku University Hospital University of Turku TE5, Hameentie 11 20521 Turku Finland. Department of Surgery, Satakunta Central Hospital, Pori, Finland; Department of Internal Medicine, University of Turku, Turku, Finland Division of Medicine, Turku University Hospital, Turku, Finland; Department of Computer Science, Aalto University, Helsinki, Finland; Faculty of Medicine, Turku University Hospital, University of Turku, Turku, Finland Department of Surgery, Satakunta Central Hospital, Pori, Finland Department of Vascular Surgery, Turku University Hospital, University of Turku, Turku, Finland

**Keywords:** Peripheral artery disease, chronic limb threatening ischemia, diabetes mellitus, revascularization, endovascular, open surgery bypass, amputation, tibial atherosclerosis, crural index, risk factor

## Abstract

**Background and objective:**

Diabetes mellitus associates with poor outcomes in chronic limb threatening ischemia but data on different hypoglycemic regimens and outcomes are lacking. We analyzed insulin-treated diabetes mellitus, non-insulin-treated diabetes mellitus, and patients without diabetes mellitus.

**Methods:**

All patients with peripheral artery disease and/or diabetes mellitus and infrapopliteal revascularization in the Department of Vascular Surgery, Turku University Hospital during 2007–2015 were included. Tibial atherosclerosis was categorized into crural index classes of I–IV.

**Results:**

Of the 497 patients, 180 were insulin-treated diabetes mellitus, 94 non-insulin-treated diabetes mellitus, and 223 patients without diabetes mellitus groups (diabetes mellitus 55.1%). Insulin-treated diabetes mellitus was the most ill, youngest (insulin-treated diabetes mellitus—median: 72.4, interquartile range: 64.0–79.5 versus non-insulin-treated diabetes mellitus—76.0, interquartile range: 67.9–83.6 versus patients without diabetes mellitus—77.3, interquartile range: 68.5–83.7, *p* < 0.001), had the highest body mass index (insulin-treated diabetes mellitus—median: 27.7, interquartile range: 24.0–31.8 versus non-insulin-treated diabetes mellitus—26.3, interquartile range: 23.2–30.3 versus patients without diabetes mellitus—23.9, interquartile range: 21.5–26.9, *p* < 0.001), and Charlson comorbidity index (insulin-treated diabetes mellitus—65.6% versus non-insulin-treated diabetes mellitus—46.8% versus patients without diabetes mellitus—10.8%, *p* < 0.001). After endovascular revascularization, limb salvage was poorer for insulin-treated diabetes mellitus (*p* = 0.046) and non-insulin-treated diabetes mellitus groups (*p* = 0.011) compared to surgery, but not for patients without diabetes mellitus (*p* = 0.15). Patients with crural index IV in insulin-treated diabetes mellitus (*p* = 0.001) and non-insulin-treated diabetes mellitus (*p* = 0.013) had higher mortality after revascularization. Crural index IV was a risk factor for limb loss (hazard ratio: 1.37, 95% confidence interval: 1.08–1.74, *p* = 0.008).

**Conclusions:**

Limb salvage after bypass is better for insulin and non-insulin diabetics, compared to the endovascular approach. Extensive tibial atherosclerosis is an independent risk factor for limb loss. It associates with increased mortality in both insulin and non-insulin diabetics.

## Introduction

Peripheral artery disease (PAD) is an atherosclerotic occlusive disease of the arteries that affects patients worldwide.^1–3^ In the future, the prevalence of PAD will likely increase due to aging populations.^1,2,4^ Diabetes mellitus (DM) is a major risk factor for PAD.^1,5^ The severity and duration of the DM are also related to PAD.^1,5^ DM has a strong association with infrapopliteal PAD.^1^ Chronic limb threatening ischemia (CLTI), the most advanced form of lower extremity PAD, raises a risk of limb amputation and for the diabetics, the risk for limb loss is even higher.^3^ To avoid amputation, a revascularization procedure is generally considered.^6^

A revascularization of the tibial vessels can be performed either as an open surgery bypass or an endovascular intervention.^3,6^ Surgical bypass has been the traditional gold standard and is most commonly performed using the saphenous vein as a conduit.^3^ Surgical bypass has been linked to greater morbidity and mortality, but better durability compared to the endovascular technique.^6^ Due to innovations in technology over the past decades and the improved skills of the operating surgeons, endovascular therapy has become increasingly popular for patients with infrapopliteal PAD.^6,7^ However, when comparing amputation-free survival (AFS) and overall survival (OS) between the two treatments, a lack of sufficient data hinders the comparison of one technique over the other, especially among diabetics.^6,8^ The information concerning outcomes in patients with different hypoglycemic regimens remain limited.

In the previous literature, atherosclerosis of the tibial vessels has not only been linked to diabetes but also linked to particularly poor AFS and OS.^9,10^ Several different classification systems for lower extremity PAD have been created, but none of them have been able to prognosticate the survival of infrapopliteal PAD patients.^9^ The crural index (CIx) has been recently characterized to grade the extent of tibial vessel atherosclerosis, and it has been demonstrated to correlate with OS, AFS, and outcome after thrombolysis.^9–11^

The aim of this study was to evaluate AFS and OS after infrapopliteal endovascular and open surgery revascularization in patients with insulin-treated DM (IT-DM), non-insulin-treated DM (NIT-DM) and patients without DM (non-DM). Special emphasis was placed on investigating whether AFS or OS was affected by the severity of the tibial atherosclerosis.

## Materials and Methods

### Study design

This was a single-center retrospective and population-based study of 497 CLTI patients and 552 limbs with infrapopliteal revascularizations performed between 1 January 2007 and 31 December 2015 in the Department of Vascular Surgery of Turku University Hospital. The study protocol was approved by the local Ethics Committee of the Hospital District of South-West Finland. Due to the retrospective nature of the study, patients’ written informed consent was not required.

### Data processing

The data search was performed using data obtained from the electronic operations database. The inclusion criteria for the study were diagnoses of PAD and/or diabetes according to the International Statistical Classification of Diseases and Related Health Problems classification system (ICD-10, I70.2, E10.x, or E11.x) and infrapopliteal revascularization during the study period. Infrapopliteal revascularizations were performed only on limbs presenting chronic limb ischemia according to TACS II and Rutherford classification classes 4–6.^12,13^ The selection on open surgery or endovascular revascularization depended on vascular specialist treating the patient. There were no exclusion criteria regarding patients’ prior medical history. Patients entered the study at the time of the primary infrapopliteal revascularization.

### CIx

Prior to revascularization, the distribution and severity of the disease were assessed by computed tomography (CT), digital subtraction angiography (DSA), or magnetic resonance angiography (MRA). According to the imaging, the tibial atherosclerosis of each patient was classified into CIx classes I–IV. If both limbs of the patient were operated, they were uniquely and separately analyzed.

The assessment of the CIx has been primary described in a study written by Jalkanen et al. In order to obtain CIx, each of the three crural vessels were analyzed individually. Only total occlusion was analyzed and each vessel was coded by the state of atherosclerosis: No detectable or minor disease: 0; total occlusion less than 5 cm: 1; total occlusion less than 15 cm: 3; and total occlusion more than 15 cm: 4. The CIx was then assessed by a sum of these three crural vessels: if the sum was 0, the CIx was 0; if the sum was 1–3, the CIx was I; if the sum was 4–6, the CIx was II; if the sum was 7–9, the CIx was III; and if the sum was 10–12, the CIx was IV. Operated limbs were divided into subgroups by the presence of DM and CIx to compare CIx. Limbs with CIx I–III were grouped together due to their low number.^9^

### Revascularizations and amputations

The data concerning revascularizations and amputations were collected from the electronic operations database. A revascularization of the tibial vessels was performed either as an endovascular intervention or as an open surgery bypass. The treatment strategy was based on the vascular status and overall condition of the patient. Endovascular procedures were predominantly performed as percutaneous transluminal angioplasty (PTA). A stent was implanted only in the case of a dissection. Open surgery procedures were performed with bypass of the femoral or popliteal arteries to the tibial or pedal arteries. Amputations were either trans-femoral or trans-tibial and only ipsilateral amputations were counted in for the survival analysis of the limb. The information concerning minor amputations (trans-pedal or toe amputations) were collected, but they were not counted as an endpoint in the survival analysis.

### Follow-up

The follow-up data (survival time) were collected until either all-cause death of the patient or the end of the study period occurred. 31 December 2015 was considered the endpoint of this study. Information concerning the death of the participant was collected from the Finnish national death index database. For AFS, the endpoint was either major ipsilateral limb amputation or death and for OS, the clinical endpoint was all-cause mortality of the patient.

### The demographic information and comorbidities

The baseline data about age, sex, weight, height, medication, and comorbidities were retrieved from electronic patient records. Only ICD-10 coded comorbidities were eligible for analysis. Cardiovascular comorbidities were collected as follows: coronary artery disease (I20.x), myocardial infarct (I21.x), heart failure (I50.x), hypertension (I10.x), atrial fibrillation (I48.x), and dyslipidemia (E78.x). The diagnosis of DM was defined as the use of insulin or other hypoglycemic-inducing agents. The diagnosis of chronic kidney failure was defined as glomerular filtration rate (GFR) less than 45 mL/min/1.73 m^2^. The Charlson comorbidity index (CCI) was used to assess each patient.^14^

### Statistical analyses

Statistic software R version 4.0.2 (R Foundation for Statistical Computing, Vienna, Austria) was used for the data processing and for statistical analyses.^15^ Shapiro–Wilk test was used to test the normality. For the characteristics of the cohort, categorical variables were assessed by the Fisher’s exact test and the continuous variables by the Kruskal–Wallis test. As for the categorical variables, frequency and percentage were used to describe data and for the continuous data, characteristics were expressed as median and interquartile range (IQR). The Mann–Kendall trend test was used to demonstrate the possible revascularizations trends. The Kaplan–Meier and Log-rank statistics were used for the survival analyses and median survival time. IQR and 95% confidence interval (CI) were calculated according to Brookmeyer and Crowley.^16^ For the analyses of the possible risk factors, an age-adjusted Cox regression model was assessed. Only the statistically significant risk factors (*p* < 0.20) in univariate analyses were forced into the multivariate regression analysis. When performing the analyses by “R,” the following packages were used: “rms”,^17^ “survival”,^18^ “comorbidity”,^19^ “Kendall”,^20^ “readxl”,^21^ “openxlsx”,^22^ and “prodlim”.^23^ The statistical significance threshold was set at 0.05.

## Results

### Group composition

Patients were categorized into three main groups: IT-DM group, NIT-DM group, and non-DM group. The IT-DM group comprised 180 patients, NIT-DM comprised 94 patients, and non-DM group comprised 223 patients.

### Characteristics

Demographic characteristics of IT-DM, NIT-DM, and non-DM groups are presented in Table 1. The total number of the study population was 497 of which diabetics comprised 55.1%. The information concerning height and weight at the time of revascularization was available for 292 patients (58.8%) and body mass index (BMI) was calculated. In IT-DM patients, BMI was higher on average compared to NIT-DM and non-DM (IT-DM—median: 27.7, IQR: 24.0–31.8 versus NIT-DM—26.3, IQR: 23.2–30.3 versus non-DM—23.9, IQR: 21.5–26.9, *p* < 0.001)

**Table 1.** Demographics and diagnosed conditions of 497 patients who underwent infrapopliteal revascularization for CLTI in Turku University Hospital during 2007–2015.

Comorbidities	Group	IT-DM N (%, IQR)	NIT-DM N (%, IQR)	Non-DM N (%, IQR)	All	*p*
	N	180	94	223	497	
	Number of males	128 (71.1)	58 (61.7)	127 (57.0)	313	0.013
	BMI	27.7 (24.0–31.8)	26.3 (23.2–30.3)	23.9 (21.5–26.9)	25.4 (22.3–29.4)	<0.001
	Coronary artery disease	49 (27.2)	20 (21.3)	37 (16.6)	106	0.035
	Myocardial infarction	63 (35.0)	29 (30.9)	46 (20.6)	138	0.004
	Heart failure	86 (47.8)	42 (44.7)	72 (32.3)	200	0.004
	Hypertension	136 (75.6)	74 (78.7)	147 (65.9)	357	0.026
	Dyslipidemia	81 (45.0)	30 (32.9)	69 (30.9)	180	0.009
	Atrial fibrillation	61 (33.9)	36 (38.2)	93 (41.7)	190	0.276
	Chronic kidney failure	31 (17.2)	9 (9.6)	11 (4.9)	51	<0.001
CCI	1–2	11 (6.1)	5 (5.3)	112 (50.2)	128	<0.001
	3–4	51 (28.3)	45 (47.9)	87 (39.0)	183	0.004
	⩾5	118 (65.6)	44 (46.8)	24 (10.8)	186	<0.001
Medication	ACE inhibitor	75 (41.7)	44 (46.8)	69 (30.9)	188	0.012
	Statin	121 (67.2)	61 (64.9)	131 (58.7)	313	0.197
CIx	Number of limbs	203	103	246	552	
	I	25 (12.3)	9 (8.7)	15 (6.1)	49	0.070
	II	48 (23.6)	26 (25.2)	66 (26.8)	140	0.742
	III	66 (32.5)	30 (29.1)	69 (28.0)	165	0.579
	IV	64 (31.5)	37 (35.9)	94 (38.2)	195	0.334

DM: diabetes mellitus; IT-DM: insulin-treated diabetics; NIT-DM: non-insulin-treated diabetics; non-DM: patients without DM; N: number; IQR: interquartile range; BMI: body mass index; ACE inhibitor: angiotensin-converting enzyme inhibitors; CCI: Charlson comorbidity index; CIx: crural index.

The IT-DM group had higher prevalence of the following cardiovascular comorbidities: coronary artery disease (IT-DM 27.2% versus NIT-DM 21.3% versus non-DM 16.6%, *p* = 0.035), myocardial infarction (IT-DM 35.0% versus NIT-DM 30.9% versus non-DM 20.6%, *p* = 0.004), heart failure (IT-DM 47.8% versus NIT-DM 44.7% versus non-DM 32.3%, *p* = 0.004), dyslipidemia (IT-DM 45.0% versus NIT-DM 32.9% versus non-DM 30.9%, *p* = 0.009), and chronic kidney failure (IT-DM 17.2% versus NIT-DM 9.6% versus non-DM 4.9%, *p* < 0.001) than NIT-DM and non-DM. In CCI comparison, the score for diabetics was higher on average: a score of ⩾5 was the most common in IT-DM (IT-DM 65.6% versus NIT-DM 46.8% versus non-DM 10.8, *p* < 0.001). ACE inhibitors were used most commonly in NIT-DM compared to IT-DM and non-DM (IT-DM 41.7% versus NIT-DM 46.8% versus non-DM 30.9%, *p* = 0.012).

### CIx

In Table 1, the CIx distribution of the cohort is presented. Before revascularization, CIx was assessed for 549 limbs. For a total of three limbs, CIx was not calculatable. Prior to revascularization, the following scanning procedures were performed: DSA was performed for 487 (88.2%), CT for 60 (10.8%), and MRA for 2 (0.4%) limbs. In the CIx distribution comparison, the disease severity did not differ between the groups.

### Revascularizations

A revascularization was performed for 552 limbs. Unilateral revascularizations were performed for 441 (79.9%) and bilateral for 111 limbs (20.1%). The chosen revascularization method was endovascular for 231 (41.8%) and surgical for 321 (58.1%) limbs. Most of the primary endovascular revascularizations, a total of 208, were performed with PTA (90.0%). A drug-coated balloon was placed in 17 (7.4%) and stent in 6 (2.3%) cases. Of surgical revascularizations, 295 (91.9%) were performed with venous conduit and 26 (8.1%) with prosthetic material.

Both IT-DM and NIT-DM groups were more likely to receive endovascular treatment than non-DM (IT-DM 56.2% versus NIT-DM 39.8% versus non-DM 30.9, *p* < 0.001), whereas open bypass procedure was predominantly performed for non-diabetics (IT-DM 43.8% versus NIT-DM 60.2% versus non-DM 69.1%, *p* < 0.001). IT-DM patients were younger at the time of primary revascularization than their NIT-DM and non-DM counterparts (IT-DM—median: 72.4, IQR: 64.0–79.5 versus NIT-DM—76.0, IQR: 67.9–83.6 versus non-DM—77.3, IQR: 68.5–83.7, *p* < 0.001).

Of 552 limbs, 143 were repetitively revascularized (25.9%). Secondary endovascular revascularization was performed for 62 limbs (IT-DM 11.8% versus NIT-DM 5.8% versus non-DM13.0%, *p* = 0.144) and secondary bypass for 62 limbs (IT-DM 12.8% versus NIT-DM 8.7% versus non-DM 11.0%, *p* = 0.559). A total of 97 limbs underwent only one repetitive revascularization (IT-DM 18.2% versus NIT-DM 13.6% versus non-DM 18.7%, *p* = 0.496), whereas 41 limbs were treated with additional two to three revascularizations (IT-DM 7.4% versus NIT-DM 4.9% versus non-DM 8.5%, *p* = 0.489). More than three secondary revascularizations were performed for five limbs (IT-DM 0.5% versus NIT-DM 1.0% versus non-DM 1.2%, *p* = 0.719).

### Amputations

A total of 194 major amputations were performed during the study period and they were predominantly performed for IT-DM, that is, for IT-DM 91, NIT-DM 34, and non-DM 69 amputations (IT-DM 46.9% versus NIT-DM 17.5% versus non-DM 35.6%, *p* < 0.001). IT-DM patients were the youngest at the time of major amputation (IT-DM—74.0, IQR: 66.7–81.7 versus NIT-DM—82.5, IQR: 74.2–88.0 versus non-DM—81.6, IQR: 73.6–88.1, *p* < 0.001). A total of 399 minor amputations were performed and of these, 155 were for IT-DM, 73 for NIT-DM, and 171 for non-DM (IT-DM 38.8% versus NIT-DM 18.3% versus non-DM 42.9% *p* = 0.259).

### Revascularization treatments 2007–2015

**Fig. 1.**
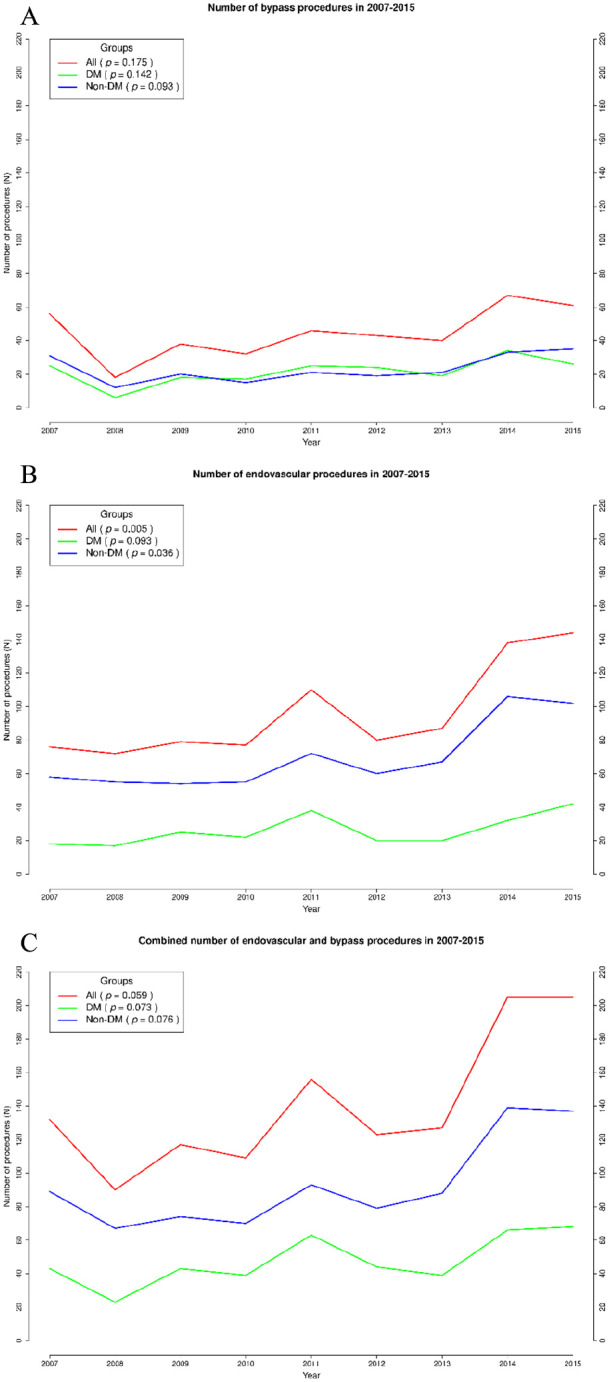
Comparisons of infrapopliteal revascularization treatments from 1 January 2007 to 31 December 2015 in the Department of Vascular Surgery of Turku University Hospital. (A) Bypass procedures. (B) Endovascular procedures. (C) Total number of revascularization procedures.

There was an increasing trend in the total amount of endovascular procedures (*p* = 0.005), as shown in Fig. 1. A similar increase in endovascular procedures was seen in the non-DM (*p* = 0.036) group. IT-DM and NIT-DM groups were combined as a DM group. The number of endovascular (*p* = 0.096) and bypass procedures (*p* = 0.142) in the DM group remained stable during the follow-up. The median for the total revascularization treatments performed per year was 127 (IQR: 117–156), of which 80 (IQR: 77–110) was for endovascular and 43 (IQR: 38–56) was for open bypasses.

### Survival analysis

The median follow-up was 25.0 months (IQR: 10.0–52.8, min 0, max 114) for IT-DM, 17.5 months (IQR: 6.0–40.0, min 0, max 106) for the NIT-DM group, and 23.0 months (IQR: 9.0–49.0, min 0, max 106) for the non-DM group. The overall 23.0 months (IQR: 9.0–48.0, min 0, max 114) was the median survival for the whole population. Baseline characteristics for IT-DM, NIT-DM, and non-DM groups and the chosen revascularization method are presented in Appendix I of Supplemental material. The corresponding information of IT-DM, NIT-DM, and non-DM groups for the severity of CIx score is presented in Appendix II of Supplemental material.

Between bypass and endovascular revascularizations, median estimated survival in months, IQR, confidence interval, and *p*-values for IT-DM, NIT-DM, and non-DM are presented in Table 2. Since the survival curve did not drop to or below 0.5 for all groups under comparison, the values of median, first, and third quartiles were not available (NA).

**Table 2.** The median estimated survival of 497 CLTI patients who underwent surgical and endovascular infrapopliteal revascularization in Turku University Hospital during 2007–2015.

IT-DM	Group	Bypass	Endovascular	*p*
AFS	Median estimated survival (IQR)	64.7 (6.8–NA)	22.0 (6.1–NA)	0.046
	95% CI for median	32.6–NA	13.0–46.4	
OS	Median estimated survival (IQR)	90.4 (17.6–NA)	NA (21.6–NA)	0.708
	95% CI for median	51.9–NA	62.0–NA	
NIT-DM	Group	Bypass	Endovascular	*p*
AFS	Median estimated survival (IQR)	NA (9.6–NA)	19.2 (1.4–NA)	0.011
	95% CI for median	48.2–NA	5.3–82.0	
OS	Median estimated survival (IQR)	NA (28.6–NA)	NA (30.8–NA)	0.096
	95% CI for median	NA–NA	NA–NA	
Non-DM	Group	Bypass	Endovascular	*p*
AFS	Median estimated survival (IQR)	90.5 (9.2–NA)	30.9 (6.5–NA)	0.15
	95% CI for median	51.4–NA	15.7–NA	
OS	Median estimated survival (IQR)	NA (24.2–NA)	64.7 (19.2–NA)	0.654
	95% CI for median	93.2–NA	30.5–NA	

DM: diabetes mellitus; IT-DM: insulin-treated diabetics; NIT-DM: non-insulin-treated diabetics; non-DM: patients without DM; AFS: amputation-free survival; OS: overall survival; IQR: interquartile range; SE: standard error; CI: confidence interval; NA: not available.

AFS was poorer after endovascular revascularization for IT-DM compared to surgical bypass (median bypass 64.7, IQR: 6.8–NA versus endovascular 22.0, IQR: 6.1–NA, *p* = 0.046). A similar difference between revascularization methods was detected for NIT-DM (median bypass NA, IQR: 9.6–NA versus endovascular 19.2, IQR: 1.4–NA, *p* = 0.011) and but not in non-DM (median bypass 90.5, IQR: 9.2–NA versus endovascular 30.9, IQR: 6.5–NA *p* = 0.15). No differences in OS were found between the procedures for the IT-DM group (median bypass 90.4, IQR: 17.6–NA versus endovascular NA, IQR: 21.6–NA, *p* = 0.708), NIT-DM group (median bypass NA, IQR: 28.6–NA versus endovascular NA, IQR: 30.8–NA, *p* = 0.096) and non-DM group (median bypass NA, IQR: 24.2–NA versus endovascular 64.7, IQR: 19.2–NA, *p* = 0.654).

A comparison between CIx I–III and CIx IV is shown in Table 3 and median estimated survival in months, IQR, confidence interval, and *p*-values are presented. Among NIT-DM (median CIx I–III: NA, IQR: 5.2–NA versus CIx IV: 12.1, IQR: 2.9–NA, *p* = 0.013) and non-DM (median CIx I–III: NA, IQR: 16.6–NA versus CIx IV: 30.9, IQR: 3.7–NA, *p* = 0.02), AFS was significantly lower than in patients with severe atherosclerosis. In IT-DM, the stage of tibial atherosclerosis did not affect limb salvage (median bypass 48.4, IQR: 6.8–NA versus endovascular 20.8, IQR: 3.6–NA, *p* = 0.114). In diabetics, both IT-DM (median bypass NA, IQR: 32.6–NA versus endovascular 42.4, IQR: 7.2–NA, *p* = 0.001) and NIT-DM (median bypass NA, IQR: 47.6–NA versus endovascular 35.9, IQR: 10.7–NA, *p* = 0.013), OS was poorer with severe tibial atherosclerosis where no such significance was seen in non-DM (median bypass NA, IQR: 30.8–NA versus endovascular NA, IQR: 28.6–NA, *p* = 0.626).

**Table 3.** The median estimated survival of CLTI patients by the severity of infrapopliteal atherosclerosis.

IT-DM	Group	CIx I–III	CIx IV	*p*
AFS	Median estimated survival (IQR)	48.4 (6.8–NA)	20.8 (3.6–NA)	0.114
	95% CI for median	20.7–82.6	12.3–43.8	
OS	Median estimated survival (IQR)	NA (32.6–NA)	42.4 (7.2–NA)	0.001
	95% CI for median	89.4–NA	21.7–90.4	
NIT-DM	Group	CIx I–III	CIx IV	*p*
AFS	Median estimated survival (IQR)	NA (5.2–NA)	12.1 (2.9–NA)	0.013
	95% CI for median	48.2–NA	8.2–70	
OS	Median estimated survival (IQR)	NA (47.6–NA)	35.9 (10.7–NA)	0.013
	95% CI for median	NA–NA	18.7–NA	
Non-DM	Group	CIx I–III	CIx IV	*p*
AFS	Median estimated survival (IQR)	NA (16.6–NA)	30.9 (3.7–NA)	0.02
	95% CI for median	64.1–NA	10.8–NA	
OS	Median estimated survival (IQR)	NA (30.8–NA)	NA (28.6–NA)	0.626
	95% CI for median	NA–NA	90.5–NA	

DM: diabetes mellitus; IT-DM: insulin-treated diabetics; NIT-DM: non-insulin-treated diabetics; non-DM: patients without DM; CIx: crural index; AFS: amputation-free survival; OS: overall survival; SE: standard error; CI: confidence interval; NA: not available.

Patients were treated with infrapopliteal revascularization in Turku University Hospital during 2007–2015.

**Fig. 2.**
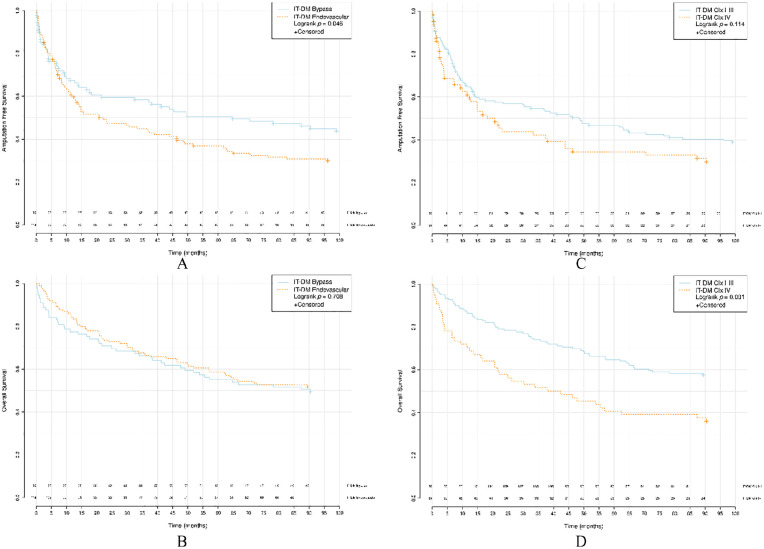
Survival analyses of IT-DM. (A) AFS between bypass versus endovascular revascularization. (B) OS between bypass versus endovascular revascularization. (C) AFS between CIx I–III versus CIx IV. (D) OS between CIx I–III versus CIx IV. IT-DM: insulin-treated diabetics; AFS: amputation-free survival; OS: overall survival; CIx: crural index.

**Fig. 3.**
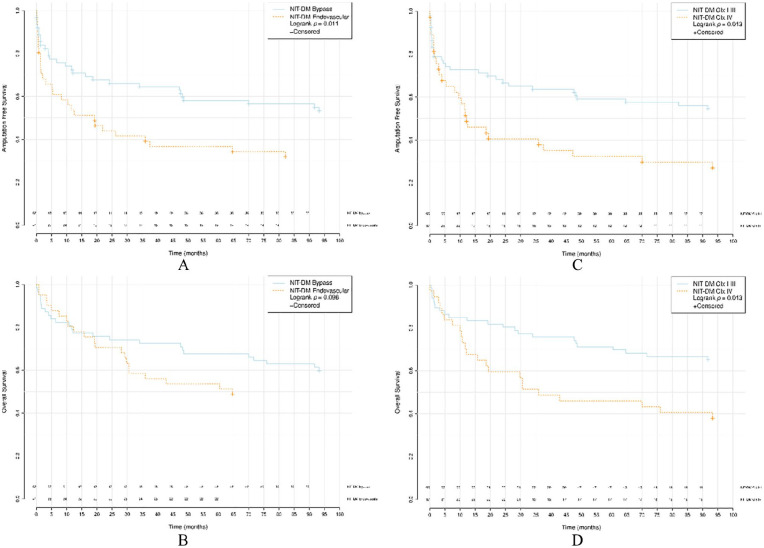
Survival analyses of NIT-DM. (A) AFS between bypass versus endovascular revascularization. (B) OS between bypass versus endovascular revascularization. (C) AFS between CIx I–III versus CIx IV. (D) OS between CIx I–III versus CIx IV. NIT-DM: non-insulin-treated diabetics; AFS: amputation-free survival; OS: overall survival; CIx: crural index.

**Fig. 4.**
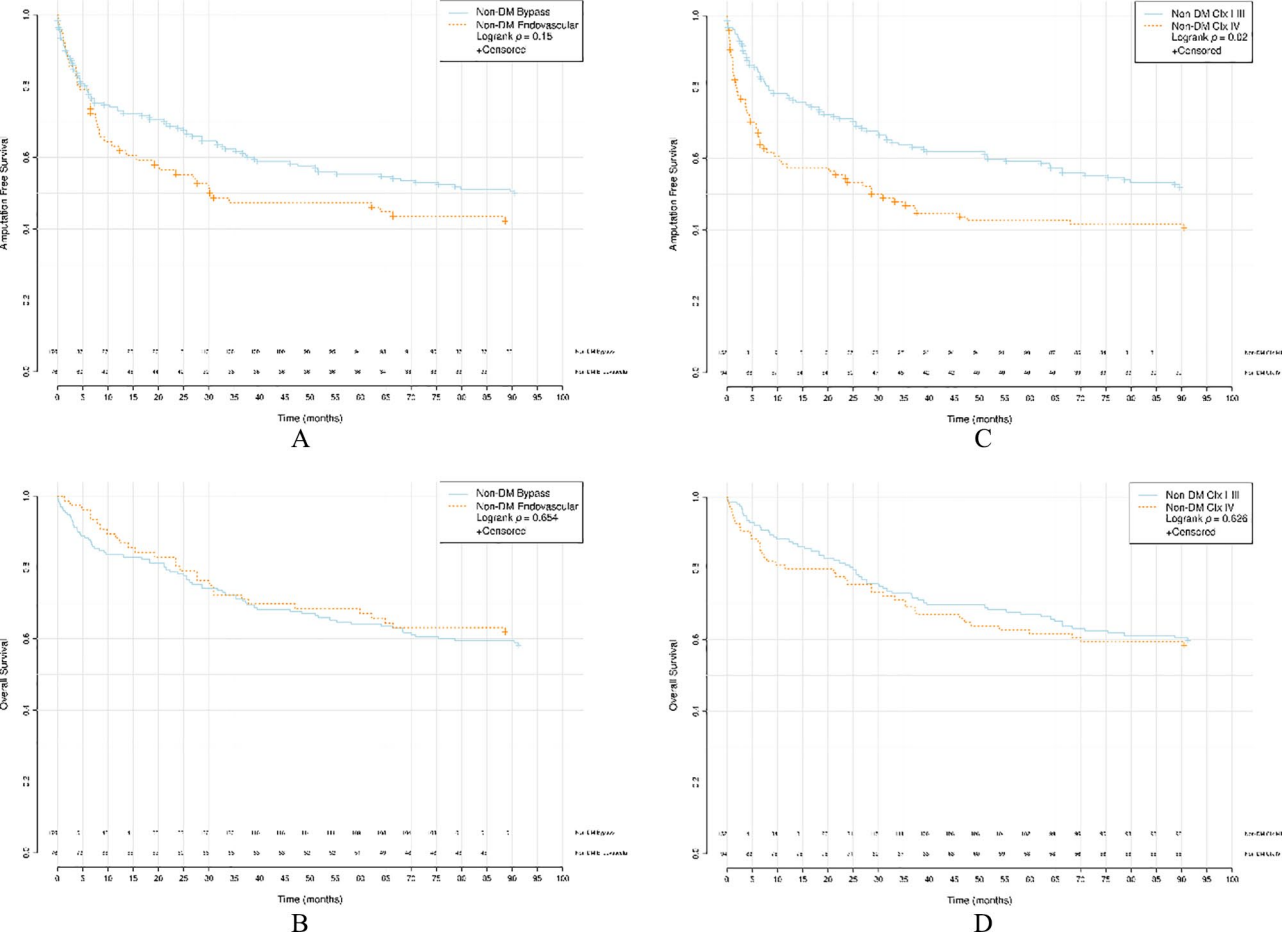
Survival analyses of non-DM. (A) AFS between bypass versus endovascular revascularization. (B) OS between bypass versus endovascular revascularization. (C) AFS between CIx I–III versus CIx IV. (D) OS between CIx I–III versus CIx IV. Non-DM: patients without DM; AFS: amputation-free survival; OS: overall survival; CIx: crural index.

Kaplan–Meier survival curves for AFS and OS in respect of groups IT-DM, NIT-DM, and non-DM between bypass and endovascular revascularization and CIx I–III and CIx IV are presented in Figs 2 to 4.

### Risk factors and survival

The OS was 81.3% at 1 year and 61.0% at 3 years and correspondingly for DM, 79.4% and 59.6% and for non-DM, 83.7% and 62.7%. For risk factor analysis, both IT-DM and NIT-DM groups were grouped together under the diagnosis of DM. According to the age-adjusted Cox regression multivariate analysis (Table 4), lower AFS was associated with advanced age (hazard ratio (HR): 1.04 per year, 95% CI: 1.02–1.05, *p* < 0.001), myocardial infarction (HR: 1.37, 95% CI: 1.04–1.81, *p* = 0.024), heart failure (HR: 1.33, 95% CI: 1.02–1.74, *p* = 0.037), and CIx IV (HR: 1.37, 95% CI: 1.08–1.74, *p* = 0.008). Better limb salvage was associated with the presence of dyslipidemia (HR: 0.63, 95% CI: 0.48–0.81, *p* < 0.001).

**Table 4.** Multivariate Cox regression model for risk factors for poor survival after infrapopliteal surgical or endovascular revascularization in the Turku University Hospital during 2007–2015.

Risk factor	AFS		OS	
	Univariate (HR, *p*)	Multivariate (HR, *p*)	Univariate (HR, *p*)	Multivariate (HR, *p*)
Age per year	1.04 (<0.001)	1.04 (<0.001)	1.04 (<0.001)	1.04 (<0.001)
DM	1.22 (0.094)	1.21 (0.182)	1.20 (0.170)	1.39 (0.043)
Coronary artery disease	1.04 (0.766)		1.35 (0.047)	1.28 (0.121)
Myocardial infarction	1.51 (0.001)	1.37 (0.024)	1.64 (<0.001)	1.39 (0.039)
Heart failure	1.70 (<0.001)	1.33 (0.037)	2.09 (<0.001)	1.60 (0.003)
Hypertension	1.04 (0.743)		0.95 (0.711)	
Dyslipidemia	0.66 (0.001)	0.63 (<0.001)	0.65 (0.002)	0.68 (0.008)
Atrial fibrillation	1.24 (0.072)	0.78 (0.053)	1.49 (0.003)	0.97 (0.825)
Chronic kidney failure	1.01 (0.945)		0.87 (0.514)	
CCI ⩾ 5	1.54 (<0.001)	1.33 (0.078)	1.43 (0.007)	0.98 (0.921)
CIx IV	1.42 (0.003)	1.37 (0.008)	1.32 (0.040)	1.26 (0.088)
BMI	1.01 (0.671)		0.98 (0.395)	

AFS: amputation-free survival; OS: overall survival; HR: hazard ratio; DM: diabetes mellitus; CCI: Charlson comorbidity index; CIx: crural index; BMI: body mass index.

Poor survival in OS analysis was associated with advanced age (HR: 1.04 per year, 95% CI: 1.03–1.06, *p* < 0.001), DM (HR: 1.39, 95% CI: 1.01–1.90, *p* = 0.043), myocardial infarction (HR: 1.39, 95% CI: 1.02–1.89, *p* = 0.039), and heart failure (HR: 1.60, 95% CI: 1.17–2.18, *p* = 0.003). Dyslipidemia was associated with a better outcome (HR: 0.68, 95% CI: 0.51–0.90, *p* = 0.008).

## Discussion

This study of 497 CLTI patients suggested that endovascular treatment was associated with worse limb salvage compared to surgical bypass for both insulin- and non-insulin-treated diabetic groups but not for patients without DM. Insulin users were the most ill of the study cohort: they were predominantly treated with endovascular revascularizations and they also underwent more amputations than non-insulin diabetics and non-diabetics. Extensive tibial atherosclerosis was an independent risk factor for limb loss and in both insulin- and non-insulin-treated diabetics, it was associated with increased mortality.

When choosing the appropriate revascularization strategy for CLTI patients, eligible evidence remains scarce. However, endovascular therapy has been increasingly popular and among many specialists, it has become the preferential option compared to traditional surgery.^6,7^ This finding in our data is also supported by earlier reviews.^6,7^ Up to this date, the bypass versus angioplasty in severe ischemia of the leg (BASIL) remains the only study that has compared the outcome of infrainguinal bypass and balloon angioplasty in patients with severe limb ischemia.^24^ A post hoc analysis found similar outcomes for AFS and OS in 30 day postoperative period but after 2 years, surgery was linked to significantly better results.^24,25^ Although BASIL provided valuable information, it did not focus, particularly, on diabetics. This implies that studies that concentrate solely on diabetics are lacking, especially with the combination of diabetes and CLTI.

### Insulin and oral-medicated diabetics

Vascular disease is usually more severe and aggressive in diabetics and the risk of amputation is significantly elevated.^26^ Most of the studies with a diabetic cohort do not have differentiated patients using the on-going hypoglycemic medication as criteria: therefore, the effect of different diabetic regimen on revascularization strategies and outcomes has not been thoroughly evaluated. Previously, insulin-treated diabetics have been demonstrated to present more severe disease at a relatively young age.^27^ In this study, the median age for insulin users at revascularization was 72.4 (IQR: 64.0–79.5), which is significantly younger than in non-insulin diabetics and non-diabetics (NIT-DM 76.0, IQR: 67.9–83.6 versus non-DM 77.3, IQR: 68.5–83.7, *p* < 0.001). In the previous literature, insulin use has been particularly associated with a multitude of comorbidities.^28^ This is analogous to the characteristics of our diabetic group on insulin: they were the subgroup that was the most ill. They also had an abundance of cardiovascular comorbidities, higher median BMI, and higher CCI scores. Similarly, our diabetics without insulin had poor baseline health compared to non-diabetics.

### Revascularizations and amputations

In our study, diabetics, both insulin and non-insulin hypoglycemic agent users, were predominantly treated endovascularly. This same treatment strategy of “endovascular first” has been reported in multiple studies and vascular centers.^27–29^ In infrapopliteal CLTI patients overall, endovascular methods can be efficient in short and stenotic lesions, whereas long occlusions may require surgical revascularization for better outcomes.^3^

After performing a bypass, superior results have been noted to be achieved with autologous vein conduit, whereas prosthetic grafts are less successful.^3,25^ Taking this into account, some studies have been performing bypasses with artificial conduits, in favor of an endovascular strategy.^25^ In addition, the experience and skill level of the operating practitioner have a major impact to the outcome, since distal bypass surgery is one of the most challenging procedures in vascular surgery.^30^

The 2017 ESC guidelines suggest that infrapopliteal lesions should be treated by surgery and with venous conduit, if it is applicable.^3^ Unfortunately, diabetics are often excluded from invasive surgery due to their poor preoperative health condition.^31,32^ A lack of sufficient venous conduit material might also exclude patients from surgical bypass. Coronary artery disease is common among diabetics with CLTI and autologous conduit might already be used for concomitant coronary bypass.^33^

According to the present observations on both insulin and non-insulin diabetics groups, endovascular intervention was more likely to precede major amputation compared to bypass. As stated before, an evident explanation is patient selection that directs these patients toward endovascular strategy. In addition, in previous literature, insulin regimen has been associated with increased limb loss: Darling et al.^27^ reported better durability with surgery compared to endovascular procedure in patients on insulin. A study conducted by Dosluoglu et al.^28^ reported that primary patency was poorer in insulin users after endovascular revascularization and insulin was associated with worse limb salvage. Rivero et al.^34^ also reported insulin usage as an independent risk factor for limb loss. Contradictory findings have also been demonstrated. For instance, a univariate analysis by Schanzer et al.^33^ did not find evidence of insulin regimen being a predictor for poor limb salvage, although a diagnosis of DM, in general, was found to be a risk factor for limb loss. The same study by Dosluoglu et al.^28^ reported that limb salvage in non-insulin diabetics was similar to that of non-diabetics.

Overall, diabetics have been noted to encounter more adverse limb events than patients without DM due to their metabolic alterations and poor glycemic control.^29^ Glucose control, in particular, has been acknowledged to have a crucial role in infrapopliteal disease and limb salvage.^3^ Considering the relatively long survival after revascularization, patients with CLTI and DM should be treated, or at least be considered for treatment by the most appropriate form of revascularization. Support for this statement comes from some studies that have reported similar OS compared to those without DM.^31,35^ Further investigations are required, although there is some evidence that refer to a benefit from open surgery compared to endovascular technique, especially for patients under insulin regimen.^35^

### CIx

The TASC II update was published in 2015 and although it provided a new, long-awaited classification for infrapopliteal PAD, it did not provide information about the most appropriate methods for the survival of these patients.^9^ Recently, the CIx was created to evaluate the severity and distribution of tibial atherosclerosis to predict survival.^9^ Extensive tibial atherosclerosis, characterized by CIx classes III and IV, has been associated with limb loss.^9,10,36^ We did not detect any difference in the CIx distribution between IT-DM, NIT-DM, and non-DM groups and interestingly, severe atherosclerosis associated with poor AFS only for NIT-DM and non-DM groups. These patients were more likely to be female (IT-DM 28.9% versus NIT-DM 38.3% versus non-DM 43.0%, *p* = 0.013), older (IT-DM 72.4, IQR: 64.0–79.5, versus NIT-DM 76.0, IQR: 67.9–83.6 versus non-DM 77.3, IQR: 68.5–83.7, *p* < 0.001) and more fragile with lower BMI (IT-DM 27.7, IQR: 24.0–31.8 versus NIT-DM 26.3, IQR: 23.2–30.3 versus non-DM 23.9, IQR: 21.5–26.9, *p* < 0.001). From these data, it could be assumed that diabetes, and also insulin use itself, do not correlate with the severity of tibial atherosclerosis and that other factors, for example, wounds and infections, rather than extensive atherosclerosis seem to expose diabetics to higher limb loss.

### Mortality

In this study, the survival was 81.3% at 1 year and 61.0% at 3 years, correspondingly for DM, 79.4% and 59.6% and for non-DM, 83.7% and 62.7%. Other studies with CLTI cohorts have reported OS rates ranging from 79.1% to 87.7% after 1-year post-operation, in concurrence with our results.^33,36,37^ A notable proportion of our CLTI patients had repetitive interventions (28.8%) and bilateral amputations (20.1%) which support the previous literature by demonstrating the vulnerability of the vascular system of this group of patients.^36^

Although the chosen primary revascularization method did not correlate with mortality, extensive atherosclerosis was associated with mortality both insulin and non-insulin diabetic. Wickström et al. (10) found that CIx III and IV score was associated with mortality, although their analysis did not focus particularly on diabetics or its subgroups as defined by medication. Accelerated alterations in the metabolic state, inflammation, endothelial dysfunction, and other proatherogenic changes that accompany diabetes might be an explanation for worse OS.^38^ Diabetes-associated peripheral neuropathy might additionally contribute to the higher mortality rate.^32^

### Risk factors

Previous studies, including BASIL, PREVENT III, and Finnvasc, found that significant cardiovascular risk factors portend to poor AFS and OS and are thus parallel with the findings of this study.^30,33,39,40^ Age, as an independent risk factor, also associated with poor outcomes.^33^ Recently, Vrsalović et al.^41^ reported a prevalence range of 8.0%–17.9% for atrial fibrillation in PAD patients and found that a combination of PAD and atrial fibrillation together associates with mortality. Interestingly, the prevalence of atrial fibrillation was strikingly high in our CLTI study population (38.3%) and supportive of Vrsalović et al.’s findings that atrial fibrillation might be a marker of severe atherosclerosis. Dyslipidemia was associated with better prognosis, most likely due to an aggressive lipid-lowering strategy as a crucial part of the treatment plan for CLTI.^3^

### Limitations

This study has some limitations. The patient selection was pre-selected before the study according to the patient characteristics and vascular status. A multitude of patient-derived factors, such as poor overall health, anesthesia risks, a lack of autologous conduit, and lesion characteristics guided the decision toward endovascular intervention. The patients with short stenosis on tibial arteries are more likely to be treated with endovascular methods. Since being a retrospective study, the data might be uncertain on comorbidities. The strengths of this study are the long follow-up periods and an adequately sized study cohort.

## Conclusion

This study proposes that open surgery bypass was associated with better limb salvage for diabetic CLTI patients, which are either insulin- or non-insulin-treated, compared to endovascular technique. Patients under an insulin regimen were the most ill of all CLTI patients and were predominantly treated with endovascular revascularizations, but they still underwent more amputations than other groups. Extensive tibial atherosclerosis was an independent risk factor for limb loss and in insulin and non-insulin diabetics, it also associated with increased mortality.

## Supplemental Material

sj-pdf-1-sjs-10.1177_1457496920968679 – Supplemental material for Chronic limb threatening ischemia and diabetes mellitus: the severity of tibial atherosclerosis and outcome after infrapopliteal revascularization Click here for additional data file. Supplemental material, sj-pdf-1-sjs-10.1177_1457496920968679 for Chronic limb threatening ischemia and diabetes mellitus: the severity of tibial atherosclerosis and outcome after infrapopliteal revascularization by Veerakaisa Koivunen, Markus Juonala, Kimmo Mikkola and Harri Hakovirta in Scandinavian Journal of Surgery

sj-pdf-2-sjs-10.1177_1457496920968679 – Supplemental material for Chronic limb threatening ischemia and diabetes mellitus: the severity of tibial atherosclerosis and outcome after infrapopliteal revascularization Click here for additional data file. Supplemental material, sj-pdf-2-sjs-10.1177_1457496920968679 for Chronic limb threatening ischemia and diabetes mellitus: the severity of tibial atherosclerosis and outcome after infrapopliteal revascularization by Veerakaisa Koivunen, Markus Juonala, Kimmo Mikkola and Harri Hakovirta in Scandinavian Journal of Surgery

## References

[1] Criqui MH Aboyans V : Epidemiology of peripheral artery disease. Circ Res 2015;116:1509–1526. 25908725 10.1161/CIRCRESAHA.116.303849

[bibr2-1457496920968679] Fowkes FGR Rudan D Rudan I , et al: Comparison of global estimates of prevalence and risk factors for peripheral artery disease in 2000 and 2010: A systematic review and analysis. Lancet 2013;382:1329–1340. 23915883 10.1016/S0140-6736(13)61249-0

[3] Aboyans V Ricco JB Bartelink MLEL , et al: 2017 ESC guidelines on the diagnosis and treatment of peripheral arterial diseases, in collaboration with the European Society for Vascular Surgery (ESVS). Eur Heart J 2018;39:763–816.

[4] Chen L Magliano DJ Zimmet PZ : The worldwide epidemiology of type 2 diabetes mellitus: Present and future perspectives. Nat Rev Endocrinol 2012;8:228–236. 10.1038/nrendo.2011.183 22064493

[5] Vrsalovic M Vucur K Vrsalovic Presecki A , et al: Impact of diabetes on mortality in peripheral artery disease: A meta-analysis. Clin Cardiol 2017;40(5):287–291. 28026025 10.1002/clc.22657 PMC6490626

[6] Jaff MR White CJ Hiatt WR , et al: An update on methods for revascularization and expansion of the TASC lesion classification to include below-the-knee arteries: A supplement to the Inter-Society Consensus for the Management of Peripheral Arterial Disease (TASC II). Vasc Med 2015;20:465–478. 26268268 10.1177/1358863X15597877

[7] Thukkani AK Kinlay S : Endovascular intervention for peripheral artery disease. Circ Res 2015;116:1599–1613. 25908731 10.1161/CIRCRESAHA.116.303503 PMC4504240

[8] Hinchliffe RJ Andros G Apelqvist J , et al: A systematic review of the effectiveness of revascularization of the ulcerated foot in patients with diabetes and peripheral arterial disease. Diabetes Metab Res Rev 2012;28:179–217. 22271740 10.1002/dmrr.2249

[9] Jalkanen JM Wickstr J Venermo M , et al: The extent of atherosclerotic lesions in crural arteries predicts survival of patients with lower limb peripheral artery disease: A new classification of crural atherosclerosis. Atherosclerosis 2016;251:328–333. 27133479 10.1016/j.atherosclerosis.2016.04.016

[bibr10-1457496920968679] Wickström JE Jalkanen JM Venermo M , et al: Crural index and extensive atherosclerosis of crural vessels are associated with long-term cardiovascular mortality in patients with symptomatic peripheral artery disease. Atherosclerosis 2017;264:44–50. 28763728 10.1016/j.atherosclerosis.2017.07.023

[11] Vakhitov D Hakovirta H Saarinen E , et al: Prognostic risk factors for recurrent acute lower limb ischemia in patients treated with intra-arterial thrombolysis. J Vasc Surg 2020;71(4):1268–1275. 31495677 10.1016/j.jvs.2019.07.061

[12] Rutherford RB Baker JD Ernst C , et al: Recommended standards for reports dealing with lower extremity ischemia: Revised version. J Vasc Surg 1997;26(3):517–538. 9308598 10.1016/s0741-5214(97)70045-4

[13] Norgren L Hiatt WR Dormandy JA , et al: Inter-society consensus for the management of peripheral arterial disease (TASC II). Eur J Vasc Endovasc Surg 2007;33:S1–S75. 17140820 10.1016/j.ejvs.2006.09.024

[14] Charlson ME Pompei P Ales KL , et al. A new method of classifying prognostic in longitudinal studies: Development. J Chronic Dis 1987;40:373–383. 3558716 10.1016/0021-9681(87)90171-8

[15] R Core Team: R: A language and environment for statistical computing, 2020, https://www.r-project.org/

[16] Brookmeyer R Crowley J : A confidence interval for the median survival time. Int Biometric Soc 1982;38:29–41.

[17] Harrell FE Jr : rms: Regression modeling strategies, 2020, https://cran.r-project.org/package=rms

[18] Therneau TM : A package for survival analysis in R, 2020, https://cran.r-project.org/package=survival

[19] Gasparini A : Comorbidity: An R package for computing comorbidity scores. J Open Source Softw 2018;3:648.

[20] McLeod AI : Kendall: Kendall rank correlation and Mann-Kendall trend test, 2011, https://cran.r-project.org/package=Kendall

[21] Wickham H Bryan J. readxl: Read Excel Files, 2019, https://cran.r-project.org/package=readxl

[22] Schauberger P Walker A : openxlsx: Read, write and edit xlsx files, 2020, https://cran.r-project.org/package=openxlsx

[23] Gerds TA : prodlim: Product-limit estimation for censored event history analysis, 2019, https://cran.r-project.org/package=prodlim

[24] Bradbury AW Adam DJ Beard JD , et al: Bypass versus angioplasty in severe ischaemia of the leg (BASIL): Multicentre, randomised controlled trial. Lancet 2005;366:1925–1934. 16325694 10.1016/S0140-6736(05)67704-5

[25] Conte MS : Bypass versus angioplasty in severe ischaemia of the leg (BASIL) and the (hoped for) dawn of evidence-based treatment for advanced limb ischemia. J Vasc Surg 2010;51(5 Suppl.):69S–75S. 20435263 10.1016/j.jvs.2010.02.001

[26] Spreen MI Gremmels H Teraa M , et al: Diabetes is associated with decreased limb survival in patients with critical limb ischemia: Pooled data from two randomized controlled trials. Diabetes Care 2016;39(11):2058–2064. 27612499 10.2337/dc16-0850

[27] Darling JD O’Donnell TFX Deery SE , et al: Outcomes after first-time lower extremity revascularization for chronic limb-threatening ischemia in insulin-dependent diabetic patients. J Vasc Surg 2018;68(5):1455–1464. 30360841 10.1016/j.jvs.2018.01.055 PMC7106939

[bibr28-1457496920968679] Dosluoglu HH Lall P Nader ND , et al: Insulin use is associated with poor limb salvage and survival in diabetic patients with chronic limb ischemia. J Vasc Surg 2010;51(5):1178–1189;discussion 1188. 20304581 10.1016/j.jvs.2009.11.077

[29] Singh S Armstrong EJ Sherif W , et al: Association of elevated fasting glucose with lower patency and increased major adverse limb events among patients with diabetes undergoing infrapopliteal balloon angioplasty. Vasc Med 2014;19:307–314. 24939930 10.1177/1358863X14538330 PMC4402094

[30] Kantonen I Lepäntalo M Luther M , et al: Factors affecting the results of surgery for chronic critical leg ischemia: A nationwide survey. J Vasc Surg 1998;27(5):940–947. 9620148 10.1016/s0741-5214(98)70276-9

[31] Hicks CW Najafian A Farber A , et al: Diabetes does not worsen outcomes following infrageniculate bypass or endovascular intervention for patients with critical limb ischemia. J Vasc Surg 2016;64:1667–1674. 27871493 10.1016/j.jvs.2016.07.107

[32] Uccioli L Meloni M Izzo V , et al: Critical limb ischemia: Current challenges and future prospects. Vasc Health Risk Manag 2018;14:63–74. 29731636 10.2147/VHRM.S125065 PMC5927064

[33] Schanzer A Mega J Meadows J , et al: Risk stratification in critical limb ischemia: Derivation and validation of a model to predict amputation-free survival using multicenter surgical outcomes data. J Vasc Surg 2008;48(6):1464–1471. 19118735 10.1016/j.jvs.2008.07.062 PMC2765219

[34] Rivero M Nader ND Blochle R , et al: Poorer limb salvage in African American men with chronic limb ischemia is due to advanced clinical stage and higher anatomic complexity at presentation. J Vasc Surg 2016;63(5):1318–1324. 27005751 10.1016/j.jvs.2015.11.052

[35] Darling JD Bodewes TCF Deery SE , et al: Outcomes after first-time lower extremity revascularization for chronic limb-threatening ischemia between patients with and without diabetes. J Vasc Surg 2018;67(4):1159–1169. 28947228 10.1016/j.jvs.2017.06.119 PMC5862717

[36] Fernandez N McEnaney R Marone LK , et al: Multilevel versus isolated endovascular tibial interventions for critical limb ischemia. J Vasc Surg 2011;54(3):722–729. 21803523 10.1016/j.jvs.2011.03.232 PMC3972254

[37] Patel SD Biasi L Paraskevopoulos I , et al: Comparison of angioplasty and bypass surgery for critical limb ischaemia in patients with infrapopliteal peripheral artery disease. Br J Surg 2016;103(13):1815–1822. 27650636 10.1002/bjs.10292

[38] Marso SP Hiatt WR : Peripheral arterial disease in patients with diabetes. J Am Coll Cardiol 2006;47:921–929. 16516072 10.1016/j.jacc.2005.09.065

[39] Bradbury AW Adam DJ Bell J , et al: Bypass versus angioplasty in severe ischaemia of the leg (BASIL) trial: A survival prediction model to facilitate clinical decision making. J Vasc Surg 2010;51(5 Suppl.): 52S–68S. 20435262 10.1016/j.jvs.2010.01.077

[40] Gray BH Grant AA Kalbaugh CA , et al: The impact of isolated tibial disease on outcomes in the critical limb ischemic population. Ann Vasc Surg 2010;24:349–359. 20045628 10.1016/j.avsg.2009.07.034

[41] Vrsalović M Presečki AV : Atrial fibrillation and risk of cardiovascular events and mortality in patients with symptomatic peripheral artery disease: A meta-analysis of prospective studies. Clin Cardiol 2017;40(12):1231–1235. 29243858 10.1002/clc.22813 PMC6490667

