# Supplementary material for: Chronic limb threatening ischemia and diabetes mellitus: the severity of tibial atherosclerosis and outcome after infrapopliteal revascularization
Source: Scand J Surg. 2020 Nov 23;110(4):472–82. doi: 10.1177/1457496920968679 (PMC8688977; doi:10.1177/1457496920968679)
Supplement: sj-pdf-1-sjs-10.1177_1457496920968679 – Supplemental material for Chronic limb threatening ischemia and diabetes mellitus: the severity of tibial atherosclerosis and outcome after infrapopliteal revascularization [file sj-pdf-1-sjs-10.1177_1457496920968679.pdf]

Appendix I.

Characteristics by DM and chosen revascularization method

In Table 1. in Appendix I, the baseline information is presented by the presence and medication for DM and the chosen revascularization method. Hypertension in the IT-DM group was more common in endovascularly treated patients (bypass 66.7% vs. endovascular 82.8%, p=0.015) and patients with CIx IV were the most commonly revascularized with bypass (bypass 40.4% vs. endovascular 24.6, p=0.022). The NIT-DM patients that had undergone endovascular procedure had heart failure (bypass 35.6% vs. 60.0%, p=0.032) and atrial fibrillation (bypass 25.4% vs. endovascular 60.0%, p=0.001) more frequently than to surgically treated patients. CIx III was more common in the NIT-DM subgroup of endovascularly treated patients (bypass 21.0% vs. endovascular 41.5%, p=0.029). The prevalence of comorbidities, medication, CCI scores, and CIx status for Non-DM did not differ between surgical and endovascular revascularization method.

Table 1. Demographics and diagnosed conditions of all 497 patients that underwent infrapopliteal revascularization for CLTI in Turku University Hospital during 2007 – 2015. Patients were distributed into groups by the presence and medication of DM and the chosen revascularization method, i.e. surgical or endovascular.

IT-DM	Group	Bypass N (%)	Endovascular N (%)	All N	*p*
	N	81	99	180	
	Limbs	89	114	203	
	Male	59 (72.8)	69 (69.7)	128	0.741
	Coronary artery disease	20 (24.7)	29 (29.3)	49	0.506
	Myocardial infarction	32 (39.5)	31 (31.3)	63	0.274
	Heart failure	35 (43.2)	51 (51.5)	86	0.296
	Hypertension	54 (66.7)	82 (82.8)	136	0.015
	Dyslipidemia	33 (40.7)	48 (48.5)	81	0.366
	Atrial fibrillation	29 (35.8)	32 (32.3)	61	0.638
	Chronic kidney failure	11 (13.4)	20 (20.2)	31	0.321
Medication	ACE-inhibitor	37 (45.7)	38 (38.3)	75	0.363
	Statin	49 (60.5)	72 (72.7)	121	0.110
CCI	1-2	7 (8.6)	4 (4.0)	11	0.226
	3-4	25 (30.9)	26 (26.3)	51	0.511
	≥5	49 (60.5)	69 (69.7)	118	0.211
CIx	I	11 (12.4)	14 (12.3)	25	1.000
	II	13 (14.6)	35 (30.7)	48	0.008
	III	29 (32.6)	37 (32.5)	66	1.000
	IV	36 (40.4)	28 (24.6)	64	0.022
NIT-DM	Group	Bypass	Endovascular		*p*
	N	59	35	94	
	Limbs	62	41	103	
	Male	41 (69.5)	17 (48.6)	58	0.051
	Coronary artery disease	12 (20.3)	8 (22.9)	20	0.799
	Myocardial infarction	20 (33.9)	9 (25.7)	29	0.491
	Heart failure	21 (35.6)	21 (60.0)	42	0.032
	Hypertension	45 (76.3)	29 (82.9)	74	0.604
	Dyslipidemia	19 (32.2)	11 (31.4)	30	1.000
	Atrial fibrillation	15 (25.4)	21 (60.0)	36	0.001
	Chronic kidney failure	5 (8.5)	4 (11.4)	9	0.723
Medication	ACE-inhibitor	30 (50.8)	14 (40.0)	44	0.393

	Statin	41 (69.5)	20 (57.1)	61	0.267
CCI	1-2	2 (3.4)	3 (8.6)	5	0.357
	3-4	33 (55.9)	12 (34.3)	45	0.055
	≥5	24 (40.7)	20 (57.1)	44	0.139
CIx	I	8 (12.9)	1 (2.4)	9	0.083
	II	19 (30.6)	7 (17.1)	26	0.165
	III	13 (21.0)	17 (41.5)	30	0.029
	IV	21 (33.9)	16 (39.0)	37	0.676
Non-DM	Group	Bypass	Endovascular		*p*
	N	160	63	223	
	Limbs	170	76	246	
	Male	94 (58.8)	33 (52.3)	127	0.453
	Coronary artery disease	27 (16.9)	10 (15.9)	37	1.000
	Myocardial infarction	34 (23.3)	12 (19.0)	46	0.854
	Heart failure	49 (30.6)	23 (36.5)	72	0.428
	Hypertension	103 (64.4)	44 (69.8)	147	0.531
	Dyslipidemia	55 (34.4)	14 (22.2)	69	0.107
	Atrial fibrillation	63 (39.4)	30 (47.6)	93	0.292
	Chronic kidney failure	5 (3.1)	6 (9.5)	11	0.079
Medication	ACE-inhibitor	52 (32.5)	17 (27.0)	69	0.520
	Statin	97 (60.6)	34 (54.0)	131	0.369
CCI	1-2	85 (53.1)	27 (42.9)	112	0.183
	3-4	61 (38.1)	26 (41.3)	87	0.761
	≥5	14 (8.8)	10 (15.9)	24	0.150
CIx	I	9 (5.3)	6 (14.6)	15	0.565
	II	48 (28.3)	18 (43.9)	66	0.534
	III	44 (25.9)	25 (61.0)	69	0.284
	IV	67 (39.4)	27 (65.9)	94	0.574

DM, diabetes mellitus; IT-DM, insulin treated diabetics; NIT-DM, non-insulin treated diabetics; Non-DM, patients without DM; N, number; ACE-inhibitor, angiotensin-converting enzyme inhibitors; CCI, Charlson Comorbidity Index; CIx, Crural Index.
