# Supplementary material for: Chronic limb threatening ischemia and diabetes mellitus: the severity of tibial atherosclerosis and outcome after infrapopliteal revascularization
Source: Scand J Surg. 2020 Nov 23;110(4):472–82. doi: 10.1177/1457496920968679 (PMC8688977; doi:10.1177/1457496920968679)
Supplement: sj-pdf-2-sjs-10.1177_1457496920968679 – Supplemental material for Chronic limb threatening ischemia and diabetes mellitus: the severity of tibial atherosclerosis and outcome after infrapopliteal revascularization [file sj-pdf-2-sjs-10.1177_1457496920968679.pdf]

Appendix II.

The cohort characteristics listed by the presence and medication for DM and severity of tibial atherosclerosis is presented in Table 2 in Appendix II. In IT-DM group, an ACE inhibitor was most commonly used for patients with severe atherosclerosis (CIx I-III 36.0% vs. CIx IV 54.5%, *p*=0.022). Moreover, patients with severe atherosclerosis were predominantly treated by surgical bypass (CIx I-III 38.3% vs. CIx IV 56.3%, *p*=0.022), whereas endovascular revascularization was more common in patients with less extensive disease (CIx I-III 61.9% vs. 43.8%, *p*=0.022). In Non-DM, men were more likely to have less severe atherosclerosis (CIx I-III 64.7% vs. CIx IV 44.8%, *p*=0.004). Coronary artery disease was more common in patients with CIx I-III (CIx I-III vs. 22.1% vs. 8.0%, *p*=0.006).

Table 2. Demographics and diagnosed conditions of all 497 patients that underwent infrapopliteal revascularization for CLTI in Turku University Hospital in 2007 – 2015. Patients were distributed into groups by the presence and medication of DM and the severity of tibial atherosclerosis (CIx).

IT-DM	Group	CIx I-III	CIx IV	All	*p*
	N	125	55	180	
	Limbs	139	64	203	
	Male	92 (73.6)	36 (65.5)	128	0.287
	Coronary artery disease	35 (28.0)	14 (25.5)	49	0.856
	Myocardial infarction	41 (32.8)	22 (40.0)	63	0.398
	Heart failure	59 (47.2)	27 (49.1)	86	0.872
	Hypertension	93 (74.4)	43 (78.2)	136	0.707
	Dyslipidemia	59 (47.2)	22 (40.0)	81	0.418
	Atrial fibrillation	41 (32.8)	20 (36.4)	61	0.733
	Chronic kidney failure	25 (20.0)	6 (10.9)	31	0.198
Medication	ACE-inhibitor	45 (36.0)	30 (54.5)	75	0.022
	Statin	85 (68.0)	36 (65.5)	121	0.734
CCI	1-2	6 (4.8)	5 (9.1)	11	0.315
	3-4	38 (30.4)	13 (23.6)	51	0.376
	≥5	81 (64.8)	37 (67.3)	118	0.865
Revascularization	Bypass	53 (38.2)	36 (56.3)	89	0.022
	Endovascular	86 (61.9)	28 (43.8)	114	0.022
NIT-DM	Group	CIx I-III	CIx IV		*p*
	N	60	34	94	
	Limbs	65	38	103	
	Male	41 (68.3)	17 (50.0)	58	0.121
	Coronary artery disease	12 (20.0)	8 (23.5)	20	0.794
	Myocardial infarction	20 (33.3)	9 (26.5)	29	0.643
	Heart failure	25 (41.7)	17 (50.0)	42	0.519
	Hypertension	47 (78.3)	27 (79.5)	74	1.000
	Dyslipidemia	20 (33.3)	10 (29.4)	30	0.819
	Atrial fibrillation	21 (35.0)	15 (44.1)	36	0.389
	Chronic kidney failure	6 (10.0)	3 (8.8)	9	1.000
Medication	ACE-inhibitor	28 (46.7)	16 (47.1)	44	1.000
	Statin	41 (68.3)	20 (58.8)	61	0.376
CCI	1-2	4 (6.7)	1 (2.9)	5	0.650
	3-4	28 (46.7)	17 (50.0)	45	0.831
	≥5	28 (46.7)	16 (47.1)	44	1.000
Revascularization	Bypass	40 (61.5)	22 (57.9)	62	0.835

	Endovascular	25 (38.5)	16 (42.1)	41	0.835
Non-DM	Group	CIx I-III	CIx IV		*p*
	N	136	87	223	
	Limbs	150	96	246	
	Male	88 (64.7)	39 (44.8)	127	0.004
	Coronary artery disease	30 (22.1)	7 (8.0)	37	0.006
	Myocardial infarction	30 (22.1)	16 (18.4)	46	0.611
	Heart failure	42 (30.9)	30 (34.5)	72	0.660
	Hypertension	93 (68.4)	54 (62.1)	147	0.385
	Dyslipidemia	42 (30.9)	27 (31.0)	69	1.000
	Atrial fibrillation	55 (40.4)	38 (43.7)	93	0.677
	Chronic kidney failure	7 (5.1)	4 (4.6)	11	1.000
Medication	ACE-inhibitor	46 (33.8)	23 (26.4)	69	0.299
	Statin	82 (60.3)	49 (56.3)	131	0.579
CCI	1-2	65 (47.8)	47 (54.0)	112	0.411
	3-4	56 (41.2)	31 (35.6)	87	0.482
	≥5	15 (11.0)	9 (10.3)	24	1.000
Revascularization	Bypass	101 (67.3)	69 (71.9)	170	0.482
	Endovascular	49 (32.7)	27 (28.1)	76	0.482

DM, diabetes mellitus; IT-DM, insulin treated diabetics; NIT-DM, not insulin treated diabetics; Non-DM, patients without DM; N, number; ACE inhibitor, angiotensin-converting enzyme inhibitors; CCI, Charlson Comorbidity Index; CIx, Crural Index.
